# Classification of bipolar disorder in psychiatric hospital. a prospective cohort study

**DOI:** 10.1186/1471-244X-12-13

**Published:** 2012-02-29

**Authors:** Terje Øiesvold, Mary Nivison, Vidje Hansen, Knut W Sørgaard, Line Østensen, Ingunn Skre

**Affiliations:** 1 Institute of Clinical Medicine, Faculty of Health Sciences, University of Tromsø, and Division of general psychiatry, Nordland Hospital, Bodø, Norway; 2 Clinic for substance abuse and specialized psychiatry, University Hospital of Northern Norway, Tromsø, Norway; 3 Institute of Clinical Medicine, Faculty of Health Sciences, University of Tromsø, and Division of general psychiatry, University Hospital of Northern Norway, Tromsø, Norway; 4 Institute of Clinical Medicine, Faculty of Health Sciences, University of Tromsø, Tromsø, Norway; 5 Division of General Psychiatry, Nordland Hospital, Bodø, Norway; 6 Department of Psychology, Faculty of Health Science, University of Tromsø, Tromsø, Norway

## Abstract

**Background:**

This study has explored the classification of bipolar disorder in psychiatric hospital. A review of the literature reveals that there is a need for studies using stringent methodological approaches.

**Methods:**

480 first-time admitted patients to psychiatric hospital were found eligible and 271 of these gave written informed consent. The study sample was comprised of 250 patients (52%) with hospital diagnoses. For the study, expert diagnoses were given on the basis of a structured diagnostic interview (M.I.N.I.PLUS) and retrospective review of patient records.

**Results:**

Agreement between the expert's and the clinicians' diagnoses was estimated using Cohen's kappa statistics. 76% of the primary diagnoses given by the expert were in the affective spectrum. Agreement concerning these disorders was moderate (kappa ranging from 0.41 to 0.47). Of 58 patients with bipolar disorder, only 17 received this diagnosis in the clinic. Almost all patients with a current manic episode were classified as currently manic by the clinicians. Forty percent diagnosed as bipolar by the expert, received a diagnosis of unipolar depression by the clinician. Fifteen patients (26%) were not given a diagnosis of affective disorder at all.

**Conclusions:**

Our results indicate a considerable misclassification of bipolar disorder in psychiatric hospital, mainly in patients currently depressed. The importance of correctly diagnosing bipolar disorder should be emphasized both for clinical, administrative and research purposes. The findings questions the validity of psychiatric case registers. There are potential benefits in structuring the diagnostic process better in the clinic.

## Background

Lifetime prevalence of bipolar spectrum disorders has been found to be between 2.6% and 5% [1]. When untreated, the illness poses high risk of morbidity and mortality [2]. There is also an increased risk of suicide compared with unipolar depression [3]. Early diagnosis of bipolar disorders may significantly reduce health care costs [4]. According to Birnbaum et al. [5] and Matza et al. [6], misdiagnosed patients received inappropriate and costly treatment regimens involving suboptimal medication treatment. An increase in psychiatric inpatient hospitalization [7] and increased acute psychiatric care services [8] are reported as well as are higher indirect costs due to work loss [5]. Correctly diagnosing bipolar disorder thus should be a priority for the health care systems both for clinical, administrative and research purposes.

Since the 1960s, psychiatric case registers have been regarded as important epidemiological research tools for estimating treated incidence, prevalence and patterns of care [9]. With the development of new and better information and communication technologies, their importance is expected to increase [10,11]. Much of the utility of a psychiatric case register, however, will depend on the validity of psychiatric diagnoses. In their review, Byrne et al. [12] conclude that relatively little high-quality work exists that systematically measures the diagnostic data validity of registers for research purposes. Almost no studies (1 out of 14) performed anything else than case note reviews to assess validity. Only two reported that the register diagnoses were blinded to the researchers and inter-rater reliability testing was performed only in three of the studies. Both studies which were blinded [13,14] in the review by Byrne et al. [12], however, concluded that the case registers were not acceptable concerning affective disorders.

Several other studies show that bipolar affective disorder is frequently misdiagnosed [15-25]. A substantial delay from symptom onset to the receipt of a bipolar diagnosis is reported [15,18,20,22]. The studies dealing with misdiagnosis have either investigated patients initially presenting with depression [16,17,23-25], or are retrospective studies on patients diagnosed with bipolar disorder [15,18-22]. Studying only patients with diagnosed depression could result in a selection bias underestimating the problem of misdiagnosing bipolar affective disorder. Recall bias is a problem in the retrospective studies.

Thus, there is a need for studies using more stringent methodological approaches to estimate the degree of misclassification. In this study a structured diagnostic interview was performed on all new patients consecutively admitted to psychiatric hospital, comparing these diagnoses with those given by the clinicians. This paper will focus on the classification of bipolar disorder as this seems to be a major challenge in the psychiatric health care system.

## Methods

### Design and participants

The North-Norwegian study on first-time admitted patients to psychiatric hospital (FINN-study) is a prospective cohort study on treated incidence, utilization, and outcome in a one-year period and a 12-month follow-up period. The University Hospital in Northern Norway in Tromsø, and Nordland Hospital in Bodø, participated. All admissions to psychiatric hospitals in a region with a population of about 500 000 people are administered by these two hospitals. There are 14 community mental health centers in the region. The psychiatric services in Northern Norway are fully described elsewhere [26].

Included in the study were patients between 18 and 65 years of age who had no previous admissions to the participating hospitals and who gave written informed consent to participate. Exclusion criteria were: Lack of language competency and cognitive impairment such as dementia, serious mental retardation or other mental incapacities preventing the individual from giving an informed written consent. Further, being discharged less than 3 days after admission, was an exclusion criterion due to The Regional Ethics Committee who required that a patient be given at least 24 hrs after admission to consider participation. This made comprehensive in/out interviews unfeasible for these short-stay patients. Of 674 first-time admitted patients, 477 patients were found eligible for participation. 272 patients gave their informed consent, and of these 250 patients (52%) with hospital diagnoses comprised the study sample.

### Data collection

Diagnoses were assessed by means of the Mini International Neuropsychiatric Interview PLUS (M.I.N.I.PLUS) [27] Norwegian version 5.0.0 [28]. M.I.N.I. was developed in Europe and USA as a short diagnostic instrument for generating DSM-IV criteria diagnoses convertible to ICD.10 diagnoses [29]. The M.I.N.I.PLUS is an extended version of the M.I.N.I. that includes information on specific phobias and has an expanded psychosis module. The M.I.N.I.PLUS is built up of 15 modules corresponding to diagnostic categories and collects information along 23 axis-I problem areas in relation to past and current symptoms. The interviews were carried out by psychiatric nurses, psychologists, graduate students in psychology, a resident doctor and a psychiatrist. Except for the two students, all had extensive clinical experience and none had therapeutic or other relations to the patients. The interviewers underwent systematic training and consecutive reliability checks using videotaped interviews. The interview was performed as soon as possible after admission when the patient was found eligible to participate in an interview and had given written consent.

An experienced psychologist (I. Skre), who in the following will be referred to as the expert, has studied the validity and reliability of psychiatric diagnoses during two decades [30,31]. The expert was not employed at the participating hospitals. She determined the diagnoses on the basis of the M.I.N.I. PLUS interviews and retrospective inspection of the patients' records. The expert was blind to the hospital diagnoses. First, the M.I.N.I.PLUS schedule, including notes made by the interviewer, was reviewed and scored according to the ICD-10 criteria as they appear in ICD-10 Diagnostic Criteria for Research [32]. In cases where the information given in the interview was meagre, lacking or contradictory, additional information about the patient was sought from the hospital records: (1) the referral letter applying for admission, which accompanies all admissions to psychiatric hospitals in Norway, (2) the notes written by the receiving medical doctor at the hospital, and (3) when involving an involuntary admission, the notes written by the specialist in psychiatry/psychology who did the formal evaluation. In order to keep the expert blind to the hospital diagnoses and the referring physician's tentative diagnosis, the information was extracted from the patient's file and read aloud to the expert by an assistant. The assistant was instructed to omit all material concerning diagnostic evaluations. The following information was extracted from these documents: (1) the symptoms and behaviour of the patient in the days and hours immediately prior to hospitalization, (2) the symptoms and behaviours observed and described by the receiving medical doctor and/or the specialist in psychiatry/psychology at the hospital. In some cases, when suspecting an organic mental disorder, any documentation of results from brain imaging and neuropsychological tests were used.

In accordance with the ICD-10, a diagnostic hierarchy was employed only when exclusion criteria were explicitly given in the diagnostic manual. When assigning more than one diagnosis, the diagnoses were listed in the following order: The first or primary diagnosis was always the disorder from which the behaviours or symptoms stemmed which had resulted in hospitalisation. Following the main diagnosis were additional disorders diagnosed in the patient, most often anxiety or somatoform disorders. Finally, diagnoses for harmful use of or dependence on psychoactive substances were assigned. Hospital clinicians are obliged to use the ICD-10 criteria and to make a diagnostic evaluation in the discharge letter which is routinely sent to the patient's GP. The hospital diagnosis is based on clinical interviews and observations made during the hospital stay. Interviews with relatives may be used as well as rating scales and structured interviews, but this is uncommon. The clinician's diagnoses are given in the discharge letter from the hospital. Usually, what is considered the main disorder causing hospitalization is entered first, as the primary diagnosis, and additional diagnoses, if given, are entered subsequently.

The Regional Ethics Committee of Northern Norway approved the study.

### Statistics

Descriptive statistics were used to present sample characteristics and the frequencies of the different diagnoses given by the clinicians as well as by the expert. Kruskall-Wallis and chi-square statistics were used to assess possible bias in the study sample. Cohen's kappa (κ) was used to estimate degree of agreement between expert and clinical diagnoses. According to the guidelines of Landis and Koch [33], a kappa agreement < .20 is poor, .21-.40 is fair, .41-.60 is moderate, .61-.80 is good and > .81 is almost perfect. SPSS 16.00 was used in the statistical analyses.

## Results

### The study sample

The study sample was comprised of 250 patients. As can be seen from Table 1 the mean age was 40.4 years, 111 (44.4%) were females, 71 (28.4%) were married or cohabiting, 241 (96.4) were of Norwegian ethnicity, 74 (29.6%) had paid work, 60 (24.0%) were voluntarily admitted and the mean length of stay was 37.1 days. Participants were younger; more often had paid work, were more often voluntarily admitted and were admitted for longer lengths of stay than nonparticipants.

**Table 1:** Characteristics and possible biases (Kruskal-Wallis & Chi-square (*X*^2^)) of the sample (N = 250)

		*Excluded* *N = 197* *(N, %)*	*Included, no participation* *N = 227* *(N, %)*	*Participated* *N = 250* *(N, %)*	*X^2^/p*
Age		41.4 (sd 21.0)	44.2 (sd 19.9)	40.4 (sd 15.2)	Kruskal-Wallis, ns
Age groups	18-39 yrs	122 (61.9%)	113 (49.8%)	139 (55.6%)	X ^2 ^= 26.64, p = .000
	40-59	33 (16.8%)	64 (28.2%)	81 (32.4%)	
	60 +	42 (21.3%)	50 (22.0%)	33 (12.0%)	
Females		92 (46.7%)	97 (42.7%)	111 (44.4%)	ns
Married		51 (26.2%)	48 (21.3%)	71 (28.4%)	ns
Norwegian ethnicity		136 (69%)	215 (94.7%)	241 (96.4%)	X^2 ^= 92.34, p = .000
Employment/income	Paid work	34 (17.3%)	50 (22.0%)	74 (29.6%)	
	National insurance benefits	121 (61.4%)	128 (56.4%)	116 (46.9%)	X^2 ^= 12.81, p = .012
	Other	42 (21.3%)	49 21.6%)	60 (24.0%)	
Voluntary admission		124 (63.6%)	138 (61.1%)	193 (78.8%)	X^2 ^= 19.89, p = .000
Length of first stay		11.6 (sd 19.6)	31.0 (sd 37.8)	37.1(sd 48.3)	Kruskal Wallis p < .001

### Degree of agreement between expert's diagnoses and clinicians' diagnoses

The expert gave a mean of 3.4 diagnoses per patient whereas the clinicians gave only 1.4. The number of diagnoses and the agreement between clinician and expert are listed in Table 2. Two main comparisons were made: any diagnosis given by clinician and expert were compared and the diagnosis listed first by the clinician or expert (primary diagnosis) was compared.

**Table 2:** Frequency and degree of agreement (Cohen's kappa/κ) between expert and clinicians regarding all diagnoses and primary diagnoses only (N = 250)

	All diagnoses					**Primary diagnoses**^**e**^				
	**N**^**a**^	**Expert** **n**^**b**^ **(%)**	**Clin**. **n**^**c**^ **(%)**	**n**^**d**^	**κ**	**N**^**a**^	**Expert** **n**^**b**^ **(%)**	**Clin**. **n**^**c**^ **(%)**	**n**^**d**^	**κ**

Organic disorders	7	6	2	1	0.24*	5	5	0	0	0
F0		(2)	(1)				(2)			
Substance use disorder	94	79	45	30	0.33*	18	4	16	2	0.18*
F10-19 (excl. F1X.5)		(32)	(18)				(2)	(6)		
Substance induced psychosis F1X.5	16	16	7	7	0.59*	13	13	7	7	0.69*
		(6)	(3)				(5)	(3)		
Schizophrenia...	40	30	32	22	0.67*	36	30	25	19	0.65*
F2		(12)	(13)				(12)	(10)		
Affective disorder	206	198	158	150	0.47*	200	190	148	138	0.45*
F3		(79)	(63)				(76)	(59)		
Bipolar disorder	56	56	18	18	0.42*	56	56	18	18	0.42*
F30-31		(22)	(7)				(22)	(7)		
Major depression	173	139	137	103	0.43*	167	133	127	93	0.41*
F32-33		(56)	(55)				(53)	(51)		
Anxiety disorder	105	100	21	16	0.15*	6	4	4	2	0.49*
F40-45 (excl. F43)		(40)	(8)				(2)	(2)		
Other	77	31	61	15	0.19*	50	4	50	4	0.12*
		(12)	(24)				(2)	(20)		

N^a ^= number of patients given the diagnosis either by expert or by clinician

n^b ^= number of patients given the diagnosis by the expert

n^c ^= number of patients given the diagnosis by a clinician

n^d ^= number of patients given the diagnosis by both the expert and a clinician

Primary diagnosis^e ^= The diagnosis considered the reason for hospitalizalization by the expert, and the diagnosis listed first in the discharge letter by the clinician

*P < 0.001

Looking at all diagnoses, affective disorder (79%) was the most common main group given by the expert, and major depression (56%) was the most common specific diagnosis. Furthermore, anxiety disorders (40%) and substance use disorders (32%) were the second and third most frequently used. Only affective disorder (63%) was common among the diagnoses determined by the clinicians. Seventy-six percent of the primary diagnoses given by the expert were in the affective spectrum (F30-F39). Both the expert and the clinicians gave very few patients (about 2%) a primary diagnosis of anxiety disorder, and only a few (2%) received a primary diagnosis of substance use disorder by the expert.

The agreement between the expert and the clinicians ranged from poor to good for the different diagnostic groups. Kappa was good only for schizophrenia and substance-induced psychosis (> 0.61). Agreement concerning affective disorder (F30-F39) was moderate, both concerning the whole spectrum, major depression and bipolar disorder (kappa values ranging from 0.41 to 0.47).

### Diagnoses given by the clinicians when the expert gave a bipolar disorder diagnosis

The expert gave 58 patients the diagnosis bipolar disorder (F 30-31). Of these only 17 (30%) were given a bipolar diagnosis by the clinicians. Almost all of the patients with mania (F30-F31) according to the expert were given the same diagnosis by the clinicians (8 out of 9). Only 6 (14%) of the 42 patients diagnosed with bipolar depression by the expert were given a diagnosis of bipolar depression by the clinician. Instead, 21 (50%) of them received a diagnosis of unipolar depression. Altogether, of the 58 patients, forty percent (N = 23) received a diagnosis of unipolar depression (F32-F33) instead of bipolar disorder.

Diagnosing bipolar depression correctly in the clinic was not influenced by the presence or absence of psychosis according to the expert (*X*^2 ^= 2.10, P = 0.16).

Fifteen patients (26%) were not given a diagnosis of affective disorder at all by the clinicians. Two of the patients were diagnosed with personality disorder (F60) by the clinicians, and 4 patients were given a primary diagnosis of substance use disorder (F10-F19).

## Discussion

76% of the primary diagnoses given by the expert were in the affective spectrum. Agreement concerning affective disorder (F30-F39) was moderate both concerning the whole spectrum, major depression and bipolar disorder. The only exception was for current mania, where clinicians correctly identified seven out of eight patients. As shown in Table 3, 16 patients (28%) of the 58 patients with a bipolar disorder did not receive an affective diagnosis (F30-F39) at all by the clinicians. This finding indicates that previous studies of patients initially presenting with depression, referred to in the Introduction, may have underestimated the problem concerning misdiagnosis of bipolar disorder. In our study, as many as 40% received a diagnosis of unipolar depression (F32-F33) instead of bipolar disorder. The most striking feature was the misdiagnosis of bipolar depression as unipolar depression (F32-F33) by the clinicians, altogether 21 patients (50%) out of 42, a finding in accordance with others [34].

**Table 3:** Primary diagnoses^f ^given by the clinicians when the expert diagnosed bipolar disorder (N = 58)

Expert		Clinicians	
Mania with psychosis F30.2, F31.2	9	Mania with psychosis F30.2, F31.2	4
		Hypomania F31.0	1
		Mania without psychosis F31.1	2
		Mania not specified F31.9	1
		Schizophrenia F22	1
Bipolar depression without psychosis F31.4	28	Bipolar depression without psychosis F31.3, F31.4	3
		Bipolar depression with psychosis F31.5	1
		Major depression without psychosis F32.0-F32.2, F33.0-F33.2	15
		Depressive episode with psychosis F32.3	1
		Other recurrent affective disorders F38.1	1
		Borderline personality disorder F60.3	1
		Substance use disorder F1	3
		Other F43.2, F50.3	3
Bipolar depression with psychosis	14	Bipolar depression without psychosis F31.4	2
		Major depression without psychosis F32.0-32.2, F33.2	3
		Major depression with psychosis F32.3, F33.3	2
		Mania with psychosis F31.2	1
		F30.3	1
		Schizoaffective depression F25.1	1
		Other F19.1, F43.2, Z00.4	4
Bipolar disorder, mixed episode F31.6	5	Bipolar depression F31.3	1
		Major depression F32-33	2
		Dependent personality disorder F60.7	1
		Observatio Z03.2	1
Bipolar disorder in remision F31.7	1	Bipolar depression F31.3	1
Bipolar disorder notspecified	1	Substance induced psychosis F15.5	1

Primary diagnoses^f ^= The diagnosis considered the reason for hospitalization by the expert, and the diagnosis listed first in the discharge letter by the clinician

The clinical consequences of underdiagnosing bipolar disorder were briefly accounted for in the introduction. Secondly, there are some potential administrative consequences of underdiagnosing bipolar disorder. Misdiagnosis can represent an undercommunication of the burden these patients constitute for the health care system and consequently give wrong indications concerning developmental strategies. Misleading medical statistics may cause spurious conclusions in planning and evaluation of treatment for patients [35].

Third, our findings indicate that register diagnoses are dubious for research purposes and this pertains especially to affective disorders, a finding which is in accordance with the two studies reviewed by Byrne et al. [12], both of which were blinded [13,14]. Further, this is in accordance with the investigations of Baca-Garcia et al. [36,37] who found diagnostic instability of psychiatric disorders in clinical practice. McConville et al. [14] conclude that the case register was not acceptable even as a screening instrument, for the diagnoses of neurotic or affective disorders.

The discrepancies found may be due to several unresolved controversies regarding the identification and classification of bipolar disorder, supposedly due to its heterogeneity [35]. There is an ongoing debate on the validity of the bipolar spectrum which could hamper both the adherence to and knowledge of bipolar disorders. Not asking for manic symptoms could also be due to the general phenomenon that clinicians rely on a limited number of heuristic principles that in some instances may lead to severe and systematic errors [38,39]. We believe clinicians are more apt to use a heuristic top-down approach when they diagnose patients, i. e. not asking for other symptoms when the patient presents with depression. The expert who uses data from a structured clinical interview, however, employs a bottom-up approach in the diagnostic process, i. e. asking questions which at first seem irrelevant. The risk of misclassification is supposedly higher using the top-down diagnostic approach in that it relies on the diagnostic manual to confirm a clinical impression rather than to openly screen for alternative or additional diagnoses. Lack of relevant information in the patients' records is shown to be a general phenomenon affecting all diagnostic groups [40].

On the other hand, diagnosing bipolar disorder is not easily ascertained due to the following reasons [25]: (1) the typical presentation of bipolar disorder, when help is sought, is usually a depressive episode; (2) the diagnostic criteria for the depressive phase of bipolar disorder and for unipolar depression are identical in ICD-10; (3) it is not easy to ascertain previous episodes of (hypo) mania by recording patient histories because subjects often consider their manic symptoms to be normal and hence do not report hypomanic episodes as symptoms. Irritable mood may be misclassified as a depressive symptom. Manic symptoms during depressive episodes are reported to be indicative of a bipolar disorder and should be given more attention [41]. There are several features of a depressive episode that could indicate that it belongs within the bipolar spectrum [42]. A probabilistic approach to develop criteria for bipolar depression has been proposed [43]. The International Society for Bipolar Disorders Diagnostic Guidelines Task Force Report proposes to distinguish between unipolar and bipolar depression in the revised versions of the DSM and ICD manuals [44]. That could raise the awareness of bipolarity in affective disorders. Further, it is shown that diagnostic irrelevant information can affect the likelihood of a diagnosis of bipolar disorder [45]. Mantere et al. [46] found in their study that no previous hospitalization, lack of psychotic symptoms and the presence of rapid cycling predicted lack of bipolar I diagnosis, while no psychotic symptoms, female gender and shorter time in treatment predicted lack of bipolar II disorder. In our study the presence or absence of psychosis did influence whether a bipolar diagnosis was given in the clinic opposed to the findings of Mantere et al. [46], but the number of patients is small (Table 3).

Our study has some advantages that strengthen the validity of the results. First, a structured diagnostic interview was performed, with additional information extracted from patients' records when necessary, and second, the clinical diagnoses were blind to the expert. On the other hand, the expert never actually saw the patient such that signs and symptoms may have been missed or misinterpreted. However, the expert only scored a symptom as present if there was given a description of overt behaviour or citations from the patient in either the interview protocol or in the hospital records. Furthermore, there is always a risk that an interview that screens for all psychiatric symptoms may be overinclusive. This possible bias may result both from a "yes-saying" response style of the patient, and from a tendency of the interviewer to put weight on positive answers about signs and symptoms that are not clinically significant. Thus, there is a risk that the high number of diagnoses given by the expert is a result of response bias and scoring bias. On the other hand, the possibility that comorbidity is not diagnosed in the clinic seems more reasonable to assume. However, we do not believe that this possible bias will disturb the main findings. Our results are in accordance with those of Pinninti et al. [47] where MINI--diagnoses were compared with clinical ones. Structured interviews are shown to be better than unstructured traditional diagnostic assessment [40,48,49], and combining structured interviewing with a review of the medical records appears to produce more accurate primary diagnoses and to identify more secondary diagnoses than routine clinical methods or a structured interview alone [49,50]. The studies reviewed by Byrne et al. [12], where only case notes were checked and no new information added, should be regarded more as reliability studies than validity studies. Additionally, the clinicians' diagnoses were blind to the expert thus avoiding bias in either direction. The interviews were made through collaboration between different professions and among them one psychiatrist. This could be a weakness. On the other hand, it reflects clinical practice in the hospitals where not all diagnoses are set by psychiatrists. Interrater reliability can be low even if diagnoses are determined by researchers as found by Cheniaux et al. [51]. However, to counter this, diagnoses were not formulated by the interviewers, but by one experienced researcher, PhD in clinical psychology, in our study.

Our study comprises only first time admissions, so the generalizability of the findings could be questioned. It can be argued that new patients are more difficult to diagnose than readmitted ones. On the other hand it is reported that a diagnosis of unipolar depression is frequently given following an initial diagnosis of bipolar disorder [7,8]. To resolve this question more studies are needed. There were some biases in the study sample. Generally the participants were younger, had more often paid work and were more often voluntarily admitted. It was expected that they were more often of Norwegian ethnicity and had longer lengths of stay in the hospital due to the inclusion criteria and the considerations of the ethics committee. We do not think these biases have affected the results of this study. There is no reason to believe that the patients not included would be more easily or correctly diagnosed in the clinic. The opposite seems more likely.

## Conclusions

Our results indicate a considerable misclassification of bipolar disorder in psychiatric hospital, mainly in patients currently depressed. The importance of correctly diagnosing bipolar disorder should be emphasized both for clinical, administrative and research purposes. These findings question the validity of psychiatric case registers. There are potential benefits in structuring the diagnostic process better in the clinic [49,52,53].

## Competing interests

The authors declare that they have no competing interests.

## Authors' contributions

TØ participated in designing the study and writing of the protocol, collected data, managed the literature searches and analyses, undertook statistical analyses and wrote the manuscript. MN participated in designing the study and writing the protocol, collected data, undertook statistical analyses and contributed to the final manuscript. LØ collected data, undertook statistical analyses and contributed to the final manuscript. VH participated in designing the study and writing of the protocol and contributed to the final manuscript. KWS participated in designing the study and writing of the protocol, collected data, undertook statistical analyses and contributed to the final manuscript. IS collected data and contributed to the final manuscript. All authors read and approved the final manuscript.

## Pre-publication history

The pre-publication history for this paper can be accessed here:

http://www.biomedcentral.com/1471-244X/12/13/prepub
